# Prevalence of dental caries in Pakistan: a systematic review and meta-analysis

**DOI:** 10.1186/s12903-021-01802-x

**Published:** 2021-09-16

**Authors:** Ammar Ahmed Siddiqui, Freah Alshammary, Mushir Mulla, Saad M. Al-Zubaidi, Eman Afroze, Junaid Amin, Salman Amin, Sameer Shaikh, Ahmed A. Madfa, Mohammad Khursheed Alam

**Affiliations:** 1 Department of Community Dentistry, College of Dentistry, Bakhtawar Amin Medical and Dental College, Multan, Pakistan; 2 Present Address: Department of Preventive Dentistry, College of Dentistry, University of Ha’il, Hail, Saudi Arabia; 3 Department of Oral and Dental Health, College of Applied Health Sciences Arrass, Qassim University, Qassim, Saudi Arabia; 4 Department of Restorative Dental Sciences, College of Dentistry, University of Ha’il, Hail, Saudi Arabia; 5 General Dental Practitioner, Private Clinic, Islamabad, Pakistan; 6 College of Applied Medical Sciences, University of Ha’il, Hail, Saudi Arabia; 7 College of Medicine and Dentistry, University of Lahore, Lahore, Pakistan; 8 Department of Oral Diagnostics, College of Dentistry, University of Ha’il, Hail, Saudi Arabia; 9 Department of Preventive Dentistry, College of Dentistry, Jouf University, Aljouf, Saudi Arabia

**Keywords:** Dental caries, Dental Decay, Prevalence, Pakistan

## Abstract

**Background:**

Optimum oral health is impossible to achieve without managing dental caries. The first step to manage dental caries at a community level is to know its prevalence and trend. Unfortunately, the prevalence of dental caries at the national/regional level is not known in many developing countries. Pakistan is no exception. The present meta-analysis was planned to document the prevalence of dental caries at the national, as well as regional level. This paper will serve as a baseline for making future health policies, and health promotion activities in the country.

**Methods:**

Literature was searched through various databases, such as PubMed, SCOPUS, and Web of science using: "Prevalence", "Dental Caries", "Dental Decay" and "Severity" as keywords. Any study that reported the prevalence of dental caries, and was conducted in the Pakistani population was included. Thirty studies fulfilled the inclusion criteria. Quality assessment of all the included studies was performed using Joanna Briggs Institute (JBI) critical appraisal checklist for prevalence studies. MedCalc software was used to analyze the data.

**Results:**

In total 27,878 subjects were included in a meta-analysis from 30 studies. The prevalence estimate of dental caries at the national level was 56.62% (95% CI: 49.54 to 63.57). The I^2^ value was 99.07% (95% CI: 98.94 to 99.18), (I^2^ > 75%) indicating heterogeneity, hence pooled proportion was reported using a random-effect model. The prevalence estimate of dental caries in Sindh was 58.946% (95% CI: 43.796 to 73.274), and in Punjab, it was 55.445% (95% CI: 44.174 to 66.44), whilst in Baluchistan and KPK combined was 51.168% (95% CI: 22.930 to 79.004).

**Conclusion:**

Based on the existing data nearly 60% of the Pakistani population have dental caries. The proportion is almost the same in all provinces. Most of the included studies were found to be of high risk.

**Supplementary Information:**

The online version contains supplementary material available at 10.1186/s12903-021-01802-x.

## Background

Oral health is a fundamental component of general health and wellbeing. Among various oral diseases, dental caries continues to affect a large number of populations around the globe even though tremendous attempts to raise awareness have been made but still the trend is on the higher side. Dental caries is recognized as a disease of ancient times. It may affect anyone, irrespective of ethnicity, age, gender, or socioeconomic status. Management of dental caries nowadays largely depends upon its risk assessment hence it is very important to map out its prevalence in any given population [1–4]. According to the World Health Organization (WHO), dental caries remains a major problem for almost every country in the world. There are many definitions of dental caries [5]. However, it is largely accepted as a multifactorial disease initiated by interactions between fermentable carbohydrates, acidogenic bacteria, and numerous host factors, comprising saliva [6, 7]. The principal cause of caries is the acid development of dietary carbohydrates that are fermented by bacteria in saliva and plaque. Possible cariogenic bacteria are usually found in relatively small amounts in healthy saliva and plaque. However, there will be a proliferation of acid-tolerant bacteria in some biological and environmental disorders, such as increased frequency of fermentable carbohydrate consumption, low pH conditions [8, 9].

Dental caries is a well-known burden on health. Untreated carious lesions can be painful and may lead to functional limitation, as well as disability [10, 11]. While dental caries is mostly preventable, the occurrence of dental caries amongst adults is high, affecting almost 35% of the world's population, making it the most predominant health condition around the world [12]. Dental caries, along with periodontal diseases are a well-known cause of tooth loss, and in some cases even edentulism causing major functional limitation, and impairment [13–15]. As a result, dental caries has long been a worldwide burden on oral health [5]. Not only does it affect oral health, it too harms the quality of life and overall health, particularly in low-income countries [16]. According to the WHO, 60–90% of children are affected by dental caries [17]. Dental caries affects all age groups, although children are affected to a greater extent than adults. To solve this dilemma, part of the solution is to accurately estimate the current burden in a given geographical location and prepare for robust dental education/health promotion programs. Data on the prevalence of caries is maintained in the WHO Country Area Profile Program database. There are, however, a few limitations: data for all age groups and all WHO countries are not available; and if data is available it is not regularly updated.

The extent of disease distribution offers a unique context for planning strategies and designing public health policies. A systematic review and meta-analysis are one of the most vital research methods for obtaining an accurate estimation of disease indicators in a society. In this study, a meta-analysis was planned to deliver evidence-based information based on which suitable health care strategies can be established to get a whole representation of the situation of dental caries amongst the Pakistani population.

Based on our knowledge, we did not find any national/regional level studies or any meta-analyses to report the prevalence of dental caries in Pakistan's general population. Therefore, this systematic review and meta-analysis were conducted to estimate the proportion of dental caries in the Pakistani population by using data from already published studies.

## Methods

### Search strategy

Literature in the English language was searched from January 1970 to June 2020 primarily from PubMed, Scopus, and Web of science using the following MESH Keywords: "Prevalence", "Dental Caries", "Dental Decay" and "Severity". Additional studies were sought from gray literature google scholar, and researchgate. Besides, we also explored the reference lists of identified articles to find further relevant studies. Literature was searched using various search strategies such as prevalence, severity, dental caries, and/or prevalence, severity, dental decay, Pakistan and/or dental caries, prevalence, severity, and/or dental caries, severity, prevalence, Pakistan and/or dental decay, prevalence, severity, and/or dental decay, severity, prevalence, Pakistan.

### Inclusion and exclusion criteria

We included the studies (a) that provided the prevalence of dental caries in the Pakistani population of any sex or age group. We excluded those articles that (a) did not provide the prevalence of dental caries or data from where prevalence cannot be calculated (b) did not publish in English language (c) involved review articles, case reports, book chapters, and letter.

### Selection of studies

**Fig. 1:**
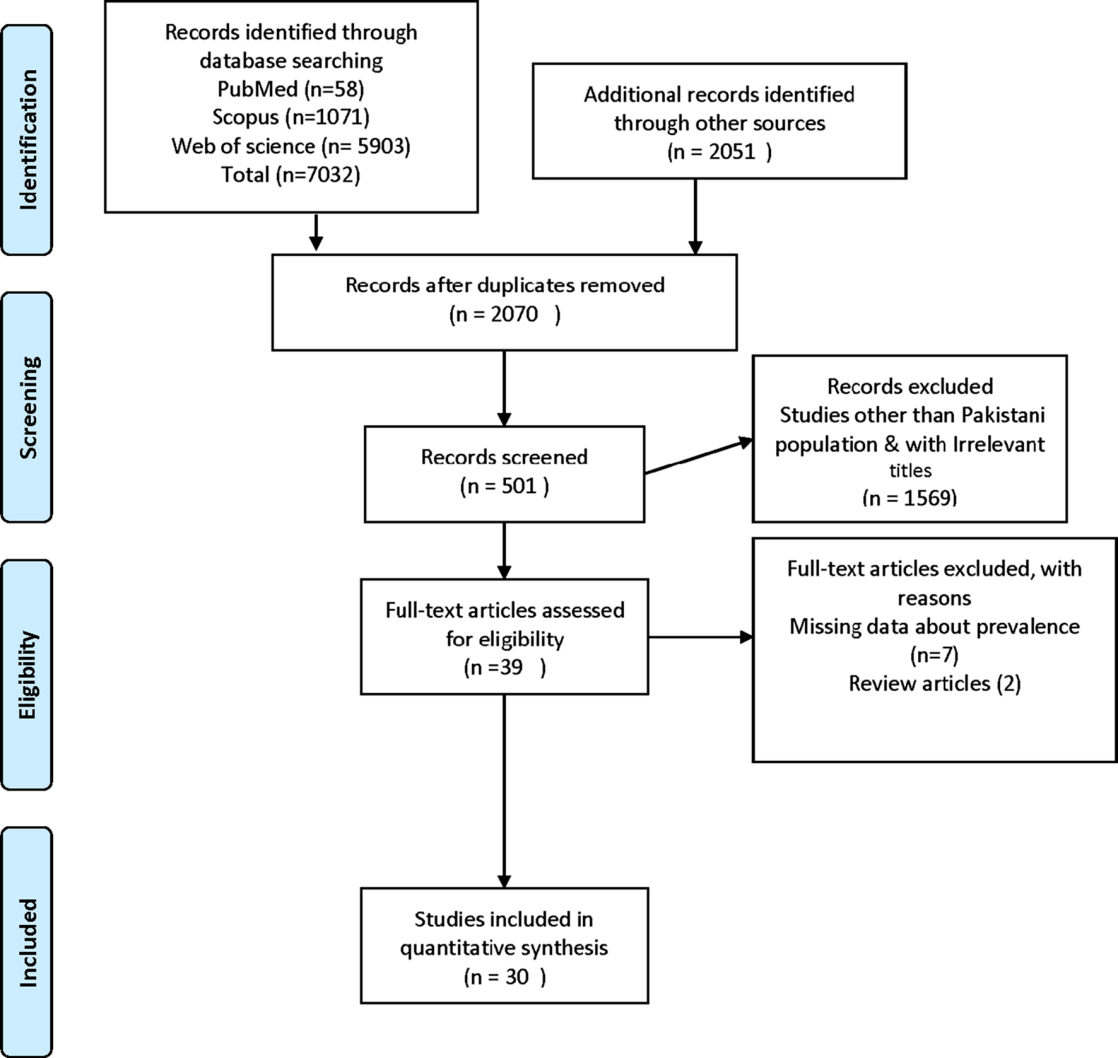
Flowchart showing selection of studies. Total number of studies found were 9083 that include from PubMed (n = 58), Scopus (n = 1071) and Web of science (n = 5903).The additional studies found through other sources were (n = 2051). The Reference Management Software Package (Endnote X9) was used to check the duplication and 7013 studies were removed. Studies (n = 1569) conducted other than Pakistani population. The remaining (n = 501) was further screened and finally (n = 39) studies was selected for full text read. Of those (n = 7) articles did not reported prevalence and (n = 2) were a review articles. Finally, (n = 30) studies were matching the objective and was satisfying the inclusion criteria for this meta-analysis and were included

The total number of studies found were 9083 that include from PubMed (n = 58), Scopus (n = 1071), and Web of Science (n = 5903). The additional studies found through other sources were (n = 2051). The Reference Management Software Package (Endnote X9) was used to check the duplication and 7013 studies were removed. Studies (n = 1569) were conducted other than the Pakistani population. The remaining (n = 501) were further screened and finally, (n = 39) studies were selected for full text read. Of those (n = 7) articles did not report the prevalence and (n = 2) were review articles. Finally, (n = 30) studies were matching the objective and were satisfying the inclusion criteria for this meta-analysis and were included (Fig. 1).

### Data abstraction

After doing the initial search, title papers and abstracts of identified articles were explored for relevance and appropriateness to the study question of the present study. The full text of the included studies was obtained. Two field-based experts (A.A.S. and E.F) independently worked on duplication and abstraction of data from each study using a standardized form. The information relating to the prevalence of dental caries, sample size, methodology, year of study, and region/ city was recorded.

### Data analysis

The pooled estimate of dental caries in Pakistan was calculated with a 95% confidence interval (CI) and data was displayed with both random-effects model and fixed-effects model. The random-effects model of the meta-analysis was considered more appropriate for the current study. In case of substantial heterogeneity among included studies, random-effects model weights study more equally and are considered more appropriate. Cochran’s Q test (χ^2^) and the I^2^ statistic were used to calculate the variance between study and heterogeneity in estimates. Cochran Q was reported as χ^2^ while I^2^ was reported in the form of percentages. A higher percentage indicated from I^2^ statistic showed high heterogeneity between estimates of individual studies (I^2^ < 25% shows low heterogeneity; 30–70% = moderate heterogeneity and > 75% shows high heterogeneity). Forest plot was used to present the combined prevalence estimate of dental caries with a 95% confidence interval (CI). The analysis was conducted by using MedCalc statistical software version 19.5.3.

### Quality assessment

**Fig. 2:**
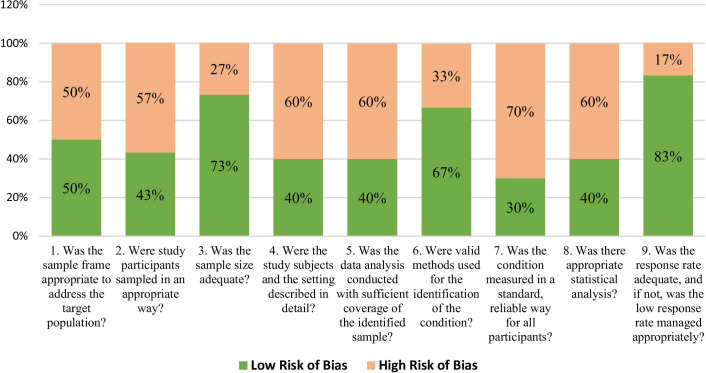
Items of JBI Critical Appraisal Checklist of included studies. JBI appraisal checklist is based on 9 items and each items is assessed by scoring (yes = 1), (no = 0), and (unclear or not applicable = 0).The total score obtained of each individual study was presented as percentages and each study was categorized according to different levels of risk of bias (high risk of bias if 20–50% items scored yes, moderate risk of bias if 50–80% items scored yes, and low risk of bias if 80–100% items scored yes as per JBI checklist)

Two independent reviewers (J.A and A.A.M) assessed the quality of included studies. Joanna Briggs Institute (JBI) critical appraisal checklist for prevalence studies was used to ascertain the risk of bias in included studies [18]. JBI appraisal checklist is based on 9 items and each item is assessed by scoring (yes = 1), (no = 0), and (unclear or not applicable = 0). The total score obtained of each study was presented as percentages and each study was categorized according to different levels of risk of bias (high risk of bias if 20–50% items scored yes, moderate risk of bias if 50–80% items scored yes, and low risk of bias if 80–100% items scored yes as per JBI checklist) as shown in Table 1 and Fig. 2.

**Table 1:**
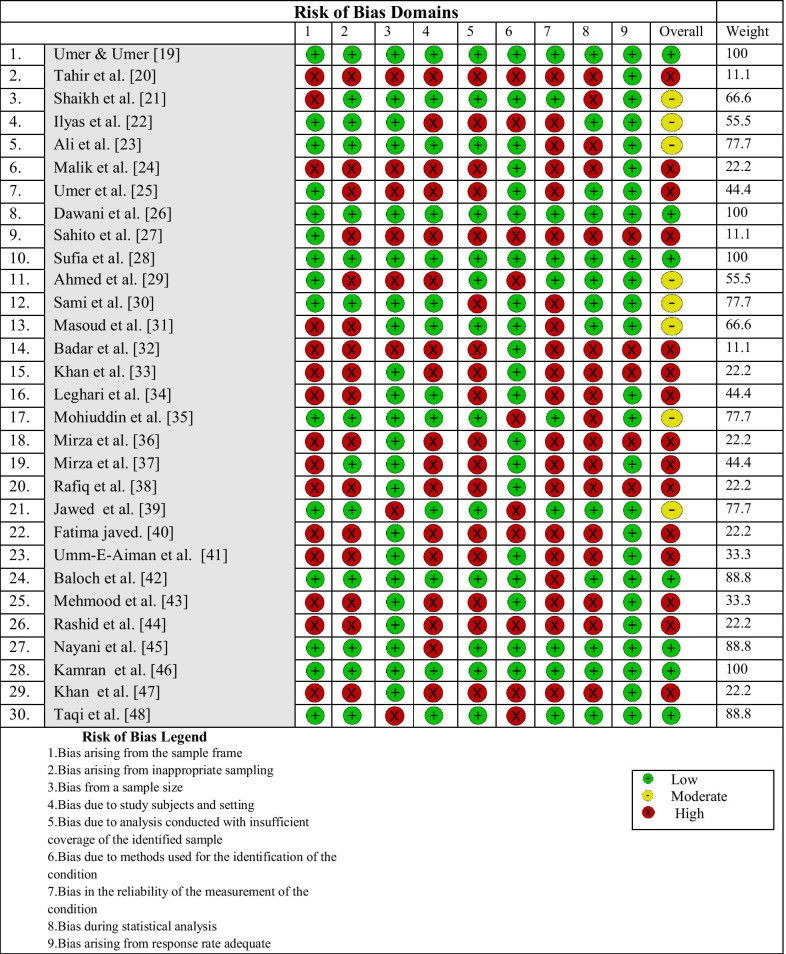
Risk of bias in the current study

## Results

A total of 27,878 subjects were included in the meta-analysis from 30 studies conducted from 2009 to 2020 on the prevalence of dental carries in Pakistan. Of those studies, 13 (43%) were from Punjab province, 11 (37%) from Sindh, 2 studies each from Khyber Pakhtunkhwa (KPK) (7%) and Baluchistan (7%), and 2 (7%) studies from Islamabad. The proportion of selected studies according to the province are classified in (Table 2). In the context of cities, there were 7 studies from Karachi, 4 from Lahore, 3 studies from Hyderabad, 2 studies each from Peshawar, Multan, Islamabad, Quetta, and Rawalpindi while one study each from Sialkot, Bahawalpur, Faisalabad, Bhakkar, Sargodha, and Khairpur city (Table 2).

**Table 2:** The characteristics of included studies in meta-analysis

Authors	Year	Age group (Years)	City/ Province	Type of dentition	Method of Caries diagnosis	Sample size (n) No. of people examined	Prevalence of dental caries (n)
Umer and Umer [19]	2011	8–10 years	Peshawar/KPK	Mixed dentition	WHO criteria	500	362
Tahir et al. [20]	2015	5–12 years	Multan/Punjab	Mixed dentition	Not reported	152	94
Shaikh et al. [21]	2014	9–18 years	Khairpur/Sindh	Permanent Dentition	WHO criteria	406	57
Ilyas et al. [22]	2015	Not reported	Hyderabad/Sindh	-	WHO criteria	278	168
Ali et al. [23]	2012	5–14 years	Lahore/Punjab	Mix dentition	WHO criteria	1673	1188
Malik et al. [24]	2014	Not reported	Karachi/ Sindh	Permanent Dentition	Caries Assessment Spectrum and treatment(CAST),third molars were not included	100	8
Umer et al. [25]	2016	3–12 years	Sargodha/Punjab	Mix dentition	Presence of Frank Cavity	518	238
Dawani et al. [26]	2012	3–6 years	Karachi/Sindh	Primary dentition	WHO criteria	1000	510
Sahito et al. [27]	2015	8–12 year	Hyderabad/Sindh	Mix dentition	Not reported	100	90
Sufia et al. [28]	2011	3–5 Years	Lahore/Punjab	Primary dentition	WHO criteria	601	243
Ahmed et al. [29]	2017	6–12 years	Hyderabad/Sindh	Mix dentition	WHO criteria	395	196
Sami et al. [30]	2016	12 years	Quetta/Baluchistan	Permanent dentition	WHO criteria	349	81
Masoud et al. [31]	2020	3–5 years	Islamabad	Primary dentition	WHO criteria	384	189
Badar et al. [32]	2012	11- 70 years	Bahawalpur/Punjab	Permanent dentition	Not reported	400	388
Khan et al. [33]	2019	12 years and above	Islamabad	Permanent dentition	Not reported	349	312
Leghari et al. [34]	2014	15 years	Karachi/Sindh	Permanent dentition	WHO criteria	392	274
Mohiuddin et al. [35]	2015	6 and 12 years	Karachi/Sindh	Mix dentition	WHO criteria	1600	1114
Mirza et al. [36]	2017	2–19 years	Lahore/Punjab	Mix dentition	WHO criteria	12,971	7409
Mirza et al. [37]	2013	3–8 years	Lahore/ Punjab	Primary dentition	WHO criteria	642	391
Rafiq et al. [38]	2019	20–80 years	Karachi/Sindh	Permanent dentition	Clinical assessment of dental caries done by DMFT	377	370
Jawed et al. [39]	2020	6–18 years	Karachi/Sindh	Mix dentition	WHO criteria	196	114
Fatima javed. [40]	2019	18–29 years	Faisalabad/Punjab	Permanent dentition	Not reported	571	74
Umm-E-Aiman et al. [41]	2018	6, 12 and 15 years age	Multan/Punjab	Mix dentition	WHO criteria	500	320
Baloch et al. [42]	2009	12 years old	Quetta/Baluchistan	Permanent dentition	Not reported	153	124
Mehmood et al. [43]	2017	5–6 years	Rawalpindi/Punjab	Primary dentition	WHO criteria	384	195
Rashid et al. [44]	2016	Not reported	Sialkot/Punjab	-	WHO criteria	1008	447
Nayani et al. [45]	2018	5–14 years	Karachi/Sindh	Mix dentition	Dental examination was done indoor by using small wooden spatula and a common hand torch light. Wooden spatula was used to retract the tongue and cheeks	500	336
Kamran et al. [46]	2017	4–17 years	Rawalpindi/Punjab	Mix dentition	WHO criteria	753	262
Khan et al. [47]	2017	12–17	Peshawar/KPK	Permanent dentition	Not reported	400	110
Taqi et al. [48]	2018	11–12 years	Bhakkar/Punjab	Mix dentition	International Caries Detection and Assessment System ICDAS	226	115

The prevalence estimate of dental caries in terms of proportion (random effect model) was 56.62% (95% CI: 49.54 to 63.57). The value of I^2^ was 99.13% (95% CI: 99.02 to 99.23) and (I^2^ > 75%) indicating high heterogeneity among the selected studies and due to this reason, aggregate data of random effect model was selected for meta-analysis. Possible reasons for the high level of heterogeneity could be because of variability in the data reported amongst the studies included in the meta-analysis. Other probable reasons may include a difference in characteristics of participants, as well as the use of the various method of caries detection, or could be because of high publication bias. The mean proportion of random and fixed effects models, along with Cochran’s Q value with *P*-value is reported in Table 3.

**Table 3:** Summary of included studies with variables and prevalence estimate of dental caries in Pakistan

Study	Sample size	Proportion (%)	95% CI	Weight (%)	
				**Fixed**	**Random**
Umer and Umer [19]	500	72.400	68.257 to 76.276	1.80	3.35
Tahir et al. [20]	152	61.842	53.620 to 69.593	0.55	3.26
Shaikh et al. [21]	406	14.039	10.810 to 17.804	1.46	3.34
Ilyas et al. [22]	278	60.432	54.417 to 66.221	1.00	3.32
Ali et al. [23]	1673	71.010	68.771 to 73.176	6.00	3.38
Malik et al. [24]	100	8.000	3.517 to 15.156	0.36	3.19
Umer et al. [25]	518	45.946	41.592 to 50.347	1.86	3.36
Dawani et al. [26]	1000	51.000	47.853 to 54.142	3.59	3.38
Sahito et al. [27]	100	90.000	82.378 to 95.100	0.36	3.19
Sufia et al. [28]	601	40.433	36.481 to 44.478	2.16	3.36
Ahmed et al. [29]	395	49.620	44.582 to 54.664	1.42	3.34
Sami et al. [30]	349	23.209	18.881 to 27.999	1.25	3.34
Masoud et al. [31]	384	49.219	44.111 to 54.339	1.38	3.34
Badar et al. [32]	400	97.000	94.818 to 98.440	1.44	3.34
Khan et al. [33]	349	89.398	85.684 to 92.425	1.25	3.34
Leghari et al. [34]	392	69.898	65.091 to 74.401	1.41	3.34
Mohiuddin et al. [35]	1600	69.625	67.306 to 71.872	5.74	3.38
Mirza et al. [36]	12,971	57.120	56.263 to 57.974	46.48	3.40
Mirza et al. [37]	642	60.903	57.008 to 64.698	2.30	3.36
Rafiq et al. [38]	377	98.143	96.212 to 99.250	1.35	3.34
Jawed et al. [39]	196	58.163	50.922 to 65.153	0.71	3.29
Fatima javed. [40]	571	12.960	10.315 to 15.995	2.05	3.36
Umm-E-Aiman et al. [41]	500	64.000	59.620 to 68.214	1.80	3.35
Baloch et al. [42]	153	81.046	73.926 to 86.923	0.55	3.26
Mehmood et al. [43]	384	50.781	45.661 to 55.889	1.38	3.34
Rashid et al. [44]	1008	44.345	41.249 to 47.474	3.62	3.38
Nayani et al. [45]	500	67.200	62.892 to 71.303	1.80	3.35
Kamran et al. [46]	753	34.794	31.391 to 38.317	2.70	3.37
Khan et al. [47]	400	27.500	23.180 to 32.157	1.44	3.34
Taqi et al. [48]	226	50.885	44.172 to 57.575	0.81	3.30
Total (fixed effects)	27,878	56.999	56.415 to 57.581	100.00	100.00
Total (random effects)	27,878	56.625	49.546 to 63.571	100.00	100.00

Q statistics = 3342.9702, DF = 29, *P* < 0.001, I^2^ = 99.13 (99.02 to 99.23)

**Fig. 3:**
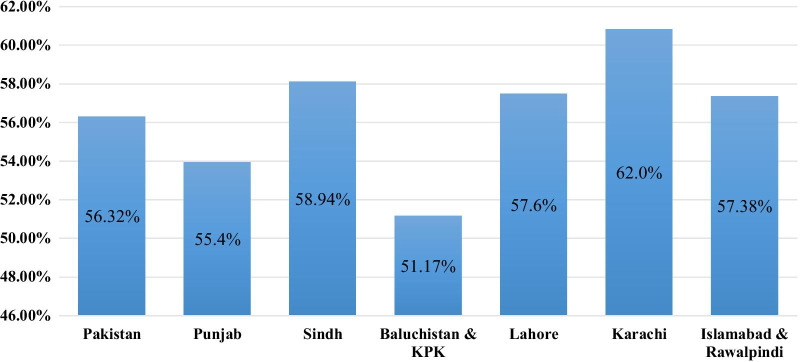
Prevalence estimate of dental caries in provinces and cities of Pakistan. The prevalence estimate (random effect model) of dental caries in Punjab was 53.95%, in Sindh 58.135% while in Baluchistan and KPK combined was 51.17% The prevalence estimate of dental caries in major cities of the countries was as following: Karachi 60.83%, Lahore 57.51%, while Islamabad and Rawalpindi combined was 57.38%

The prevalence estimate (random effect model) of dental caries in Punjab was 55.445% (95% CI: 44.174 to 66.44), in Sindh 58.946% (95% CI: 43.796 to 73.274) while in Baluchistan and KPK combined was 51.168% (95% CI: 22.930 to 79.004). The prevalence estimate of dental caries in major cities of the countries was as following: Karachi 61.988% (95% CI: 45.504 to 77.161), Lahore 57.604% (95% CI: 47.727 to 67.183), while Islamabad and Rawalpindi combined was 57.377% (95% CI: 32.642 to 80.287). The prevalence estimate of different provinces and cities of Pakistan is shown in Fig. 3.

The proportion estimate was calculated for the primary, mixed and permanent dentition as shown in the additional files (Additional file 1: Tables S1, Additional file 2: S2, Additional file 3: S3 and Additional file 4: Figure S1, Additional file 5: Fig. S2). The prevalence estimate (random effect model) of dental caries in primary dentition was 50.493% (95% CI: 43.867 to 57.110), in mixed dentition 61.183% (95% CI: 43.796 to 73.274) while in permanent dentition was 57.184% (95% CI: 26.288 to 85.251).

**Fig. 4:**
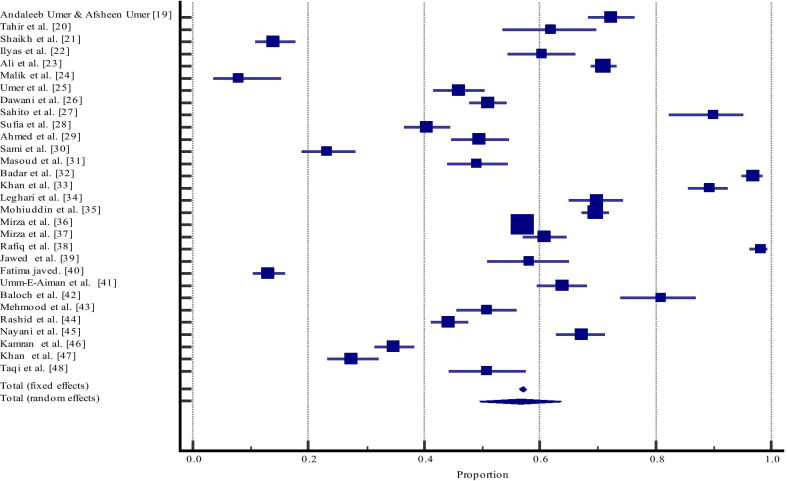
Forest plot showing effect of different studies and overall effect at 95% CI regarding dental caries from (2009–2020). Forest plot is displaying the proportion prevalence of dental caries of each study included in meta-analysis. The highest prevalence of dental caries was reported by Badar et al. in Bahawalpur while lowest was reported by Malik et al. in Karachi

The Forest plot (Fig. 4) is displaying the proportion prevalence of dental caries of each study included in the meta-analysis. The highest prevalence of dental caries was reported by Badar et al. [32] in Bahawalpur while the lowest was reported by Malik et al. [24] in Karachi.

**Fig. 5:**
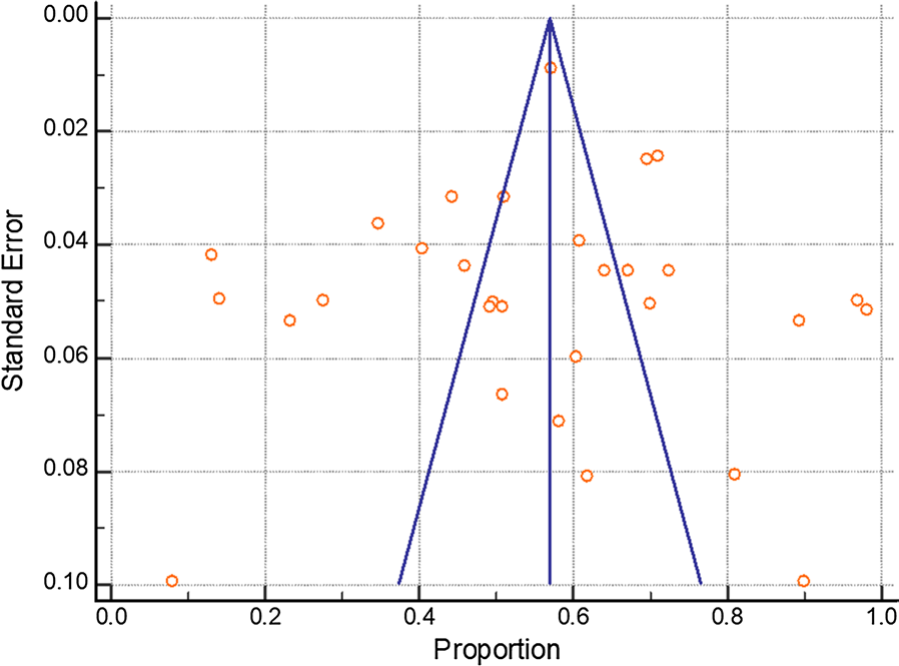
Funnel plot showing prevalence of dental caries as proportion. The funnel plot shows the effect estimates of the included studies against their measure of precision or size of the studies. The funnel plot is showing asymmetry that is indicating heterogeneity and reporting bias

The funnel plot (Fig. 5) shows the effect estimates of the included studies against their measure of precision or size of the studies. The funnel plot is showing asymmetry that is indicating heterogeneity and reporting bias. Moreover, poor methodological design and studies with smaller sample sizes can also lead to asymmetry. Other than the aforementioned reasons, the additional likelihood of asymmetry could be due to language bias (reporting of study in English language only) and citation bias (in which positive outcomes are used more to cite and readily available in scientific databases).

## Discussion

The present study concentrated on all the articles reporting the prevalence of dental caries among a Pakistani population. Thirty studies met the inclusion requirements and were included in this systematic review. Prevalence of dental caries was displayed overall, as well as for primary, mixed and permanent dentition separately. Most of the included studies were of high risk, and some of them did not mention the age groups of the participant or even the method used for detecting dental caries. Within all mentioned limitations to our best knowledge, this is the first meta-analysis on dental caries for the Pakistani population. It will help in providing a proportion estimate of dental caries for the Pakistani population. Additionally, it is indicated that most of the studies on dental caries in a Pakistani population contain a high level of bias. Future studies should be carefully designed.

Even though the current research reported useful information in terms of prevalence and seriousness of dental caries in Pakistani individuals, it is clear that most of the studies were conducted in Punjab and Sindh, with some studies conducted in Baluchistan, KPK, and Islamabad. The present meta-analysis, however, may not be indicative of the population as a whole. It may, however, be argued that there are similar socioeconomic and cultural backgrounds among the participants.

The utilize of numerous methodologies such as diagnosis, sample size, and recording procedures, randomization, and form of study was another potential weakness that is typical in dental caries studies. Heterogeneity and publication bias are other inevitable shortcomings of most meta-analysis research, which was also evident in the current meta-analysis. We used Cochran’s Q test (χ^2^) and the I^2^ statistic for verifications: the funnel plots showed asymmetrical shape at the bottom in prevalence studies indicating the presence of publication bias, which was confirmed by the insignificant result of Cochran’s Q test (χ^2^) and the I^2^ statistic.

By visual inspection of the forest plot, heterogeneity can be estimated. Where there is a low correlation in confidence intervals for the outcomes of individual studies, this usually indicates the existence of statistical heterogeneity [49]. Therefore, we used a random-effects model for the calculation of proportion estimate of dental caries, While the confidence interval quantifies the precision of the point estimate, the true dispersion of effect sizes is discussed by the prediction interval. Two problems are unique and not synonymous. Therefore, we should also estimate the prediction interval if we use a random-effects model to make inferences that are more insightful in meta-analyses [50].

DMFT index is the most used index for the measurement of dental caries at the population level. According to Castro et al. [51], most of the study participants thought to use some other index yet continued to use it as according to them, they could not found a more reliable method of measurement of dental caries. Almost all indices have limitations. To date, DMFT is a widely used and accepted method of measuring dental caries at the community level. It can only detect cavitated lesions and cannot account for incidence [52].

The overall quality of evidence in the selected studies was classified as moderate, with the majority of the studies achieving a moderate risk of bias. Seven studies were found to have low risk. In the present meta-analysis study, the prevalence of dental caries in primary, mixed and permanent teeth studied in this study. In the primary dentition, the prevalence of caries was 50.493%, and mixed dentition was 61.183%, whereas it was around 57.184% of the permeant dentition. During the start of 1980s The World Health Organization alongside FDI World dental federation formulated goals to control the spread of dental caries and mentioned that 50% of children between the ages of 5 and 6 should be free of dental caries by the year 2000 [53]. However, till date in most countries, the prevalence of dental caries in children reported to be very high. This situation creates troubling conditions for tooth decay in adulthood and would also place enormous costs for tooth restoration on the health sector in the country. The overall prevalence estimate of the proportion of dental caries (random effect model) was 56.32%. The identified factors for dental caries are poor oral hygiene habits, intake of cariogenic diet, and low socioeconomic status [54]. The above findings demonstrated high levels of both prevalence and severity in terms of caries. In various included studies, the prevalence of dental caries was reported to be varied. This is in agreement with the finding of Richardson et al. [55] that reported the frequency of dental caries in various studies differs significantly, because of many factors, including (1) subjects studied; their age, and the accessibility for examination; (2) racial and cultural factors; (3) socio-economic status; and (4) diagnostic criteria. Also, the prevalence of dental caries is typically incomparable with another in one region, so it is not possible to extrapolate findings from one ethnic group within that group [55].

As a result of many clinical studies and preventive initiatives focused on caries prevention, developed countries have less caries prevalence and a decrease in caries levels in contrast to countries with good oral health systems such as the Scandinavian countries, dental caries is still a continuing oral health issue [56]. There exists a continuous need of measuring the incidence/prevalence of dental caries. The findings of the 2013 Child Dental Health Survey in England, Wales, and Northern Ireland showed that the prevalence of caries was 31% in five-year-old kids [57]. Treatment needs for dental caries depend upon the changing pattern of a disease over time. A study from the United States reported that the prevalence of dental caries in school-going children was low since the 1960s, however, the incidence seems to be slightly increased from 24 to 28% during the late 1980s to 2004. [58]. That is why regular monitoring of disease prevalence's overtime is of essential importance. A study on 2214 Australian children aged 5 to 8 years reported the prevalence of dental caries to be lower than the current pooled prevalence of 56.32% [59].

Generally, the prevalence of dental caries in the current study was 56.32%. There were high differences within the included studies with the lowest of 8% stated by Malik et al. [24] and the highest of 97% exhibited by Badar et al. [32]. A generally low level of reported prevalence can be because of widespread usage of fluoridated toothpaste [60] and the introduction of a national oral health program [61]. Other probable reasons for such variance can be due to the various geographical areas, the variations between the individuals included in the analysis, and sample size. Oral health policies, fluoridation of community water, and oral hygiene products often play a role in the variability between countries [62]. In most provinces of Pakistan, low levels of water fluoridation were observed, likewise, only 22 percent of the Libyan population receives fluoridated water [63]. Consumption of foods containing sugar is high and easily available everywhere like schools, offices in Pakistan which can be one of the probable causative factors for a higher rate of dental caries in the country.

Some remarkable points were noted during the quality evaluation, which should be considered for future studies by researchers. More specifically, the occurrence of caries in deciduous and permanent dentition should be reported separately. Along with the mean prevalence of the age groups included in the report, the prevalence of caries for individual ages should also be indicated.

The present meta-analysis found studies with certain methodological flaws such as sampling technique, sample size. Besides that, we also noticed a strong publication bias. Another probable limitation observed was the geographical distribution of studies that contain data on prevalence was mainly reported from larger cities of the country. A substantial region of Pakistan is still unexposed, and there still can be an unexplained prevalence of dental caries. It could therefore be assumed that the findings obtained could not present an accurate picture of the prevalence of dental caries in Pakistan. There is a need for national-level population-based studies with equal representation from urban and rural areas of the country. In addition, future epidemiological studies should be conducted to explore various determinant factors of dental caries in the countries. It will help the policymaker in managing the burden of dental caries in the Pakistani population.

## Conclusions

Within the limitations of this study, it can be concluded that in Pakistan dental caries is a serious dental public health issue. Dental caries in Pakistan was found to be approximately 60%. Most of the studies on dental caries are of poor quality and contain a high amount of bias. To get a precise image of the prevalence of dental caries amongst subjects in the area, additional studies documenting dental caries from all cities are needed.

Therefore, in Pakistan, the level of dental caries should be a priority, and oral health care investment should be devoted to the preparation of oral health policies and programs. That will enhance the oral health-related quality of life of this demographic part.

## Supplementary Information

**Additional file 1**. **Table S1:** Prevelence of dental caries in primary dentition.

**Additional file 2**. **Table S2:** Prevelence of dental caries in mixed dentition.

**Additional file 3**. **Table S3:** Prevelence of dental caries in permanent dentition.

**Additional file 4**. **Table S4:** Forest plot for subgroup analysis.

**Additional file 5**. **Table S5:** Funnel plot for subgroup analysis.

## Data Availability

All data analyzed during this study are included in this manuscript.

## Abbreviations

WHO: World Health Organization
CI: Confidence interval
JBI: Joanna Briggs Institute
KPK: Khyber Pakhtunkhwa
