# Supplementary material for: Prevalence of dental caries in Pakistan: a systematic review and meta-analysis
Source: BMC Oral Health. 2021 Sep 16;21:450. doi: 10.1186/s12903-021-01802-x (PMC8447584; doi:10.1186/s12903-021-01802-x)
Supplement: Supplementary file 1 — Additional file 1. Table S1: Prevelence of dental caries in primary dentition. [file 12903_2021_1802_MOESM1_ESM.docx]

**Table S1.** Summary of included studies with variables and prevalence estimate of dental caries in primary dentition

| **Study** | **Sample size** | **Proportion (%)** | **95% CI** | **Weight (%)** | |
| --- | --- | --- | --- | --- | --- |
|  |  |  |  | **Fixed** | **Random** |
| Dawani et al. [26] | 1000 | 51.000 | 47.853 to 54.142 | 33.19 | 20.78 |
| Sufia et al. [28] | 601 | 40.433 | 36.481 to 44.478 | 19.96 | 20.18 |
| Masoud et al. [31] | 384 | 49.219 | 44.111 to 54.339 | 12.77 | 19.38 |
| Mirza et al. [37] | 642 | 60.903 | 57.008 to 64.698 | 21.32 | 20.27 |
| Mehmood et al. [43] | 384 | 50.781 | 45.661 to 55.889 | 12.77 | 19.38 |
| Total (fixed effects) | 3011 | 50.753 | 48.952 to 52.552 | 100.00 | 100.00 |
| Total (random effects) | 3011 | 50.493 | 43.867 to 57.110 | 100.00 | 100.00 |
