# Supplementary material for: Prevalence of dental caries in Pakistan: a systematic review and meta-analysis
Source: BMC Oral Health. 2021 Sep 16;21:450. doi: 10.1186/s12903-021-01802-x (PMC8447584; doi:10.1186/s12903-021-01802-x)
Supplement: Supplementary file 2 — Additional file 2. Table S2: Prevelence of dental caries in mixed dentition. [file 12903_2021_1802_MOESM2_ESM.docx]

**Table S2.** Summary of included studies with variables and prevalence estimate of dental caries in mixed dentition

| **Study** | **Sample size** | **Proportion (%)** | **95% CI** | **Weight (%)** | |
| --- | --- | --- | --- | --- | --- |
|  |  |  |  | **Fixed** | **Random** |
| Andaleeb Umer & Afsheen Umer [19] | 500 | 72.400 | 68.257 to 76.276 | 2.49 | 7.83 |
| Tahir et al. [20] | 152 | 61.842 | 53.620 to 69.593 | 0.76 | 7.17 |
| Ali et al. [23] | 1673 | 71.010 | 68.771 to 73.176 | 8.33 | 8.05 |
| Umer et al. [25] | 518 | 45.946 | 41.592 to 50.347 | 2.58 | 7.84 |
| Sahito et al. [27] | 100 | 90.000 | 82.378 to 95.100 | 0.50 | 6.75 |
| Ahmed et al. [29] | 395 | 49.620 | 44.582 to 54.664 | 1.97 | 7.74 |
| Mohiuddin et al. [35] | 1600 | 69.625 | 67.306 to 71.872 | 7.97 | 8.05 |
| Mirza et al. [36] | 12971 | 57.120 | 56.263 to 57.974 | 64.55 | 8.14 |
| Jawed et al. [39] | 196 | 58.163 | 50.922 to 65.153 | 0.98 | 7.37 |
| Umm-E-Aiman et al. [41] | 500 | 64.000 | 59.620 to 68.214 | 2.49 | 7.83 |
| Nayani et al. [45] | 500 | 67.200 | 62.892 to 71.303 | 2.49 | 7.83 |
| Kamran et al. [46] | 753 | 34.794 | 31.391 to 38.317 | 3.75 | 7.93 |
| Taqi et al. [48] | 226 | 50.885 | 44.172 to 57.575 | 1.13 | 7.47 |
| Total (fixed effects) | 20084 | 59.058 | 58.374 to 59.739 | 100.00 | 100.00 |
| Total (random effects) | 20084 | 61.183 | 55.150 to 67.049 | 100.00 | 100.00 |
