# Supplementary material for: Prevalence of dental caries in Pakistan: a systematic review and meta-analysis
Source: BMC Oral Health. 2021 Sep 16;21:450. doi: 10.1186/s12903-021-01802-x (PMC8447584; doi:10.1186/s12903-021-01802-x)
Supplement: Supplementary file 3 — Additional file 3. Table S3: Prevelence of dental caries in permanent dentition. [file 12903_2021_1802_MOESM3_ESM.docx]

**Table S3.** Summary of included studies with variables and prevalence estimate of dental caries in permanent dentition

| **Study** | **Sample size** | **Proportion (%)** | **95% CI** | **Weight (%)** | |
| --- | --- | --- | --- | --- | --- |
|  |  |  |  | **Fixed** | **Random** |
| Shaikh et al. [21] | 406 | 14.039 | 10.810 to 17.804 | 12.52 | 12.50 |
| Sami et al. [30] | 349 | 23.209 | 18.881 to 27.999 | 10.76 | 12.49 |
| Badar et al. [32] | 400 | 97.000 | 94.818 to 98.440 | 12.33 | 12.50 |
| Khan et al. [33] | 349 | 89.398 | 85.684 to 92.425 | 10.76 | 12.49 |
| Leghari et al. [34] | 392 | 69.898 | 65.091 to 74.401 | 12.08 | 12.50 |
| Rafiq et al. [38] | 377 | 98.143 | 96.212 to 99.250 | 11.62 | 12.50 |
| Fatima javed. [40] | 571 | 12.960 | 10.315 to 15.995 | 17.59 | 12.51 |
| Khan et al. [47] | 400 | 27.500 | 23.180 to 32.157 | 12.33 | 12.50 |
| Total (fixed effects) | 3244 | 54.079 | 52.348 to 55.803 | 100.00 | 100.00 |
| Total (random effects) | 3244 | 57.184 | 26.288 to 85.251 | 100.00 | 100.00 |
