# Supplementary material for: Prevalence of dental caries in Pakistan: a systematic review and meta-analysis
Source: BMC Oral Health. 2021 Sep 16;21:450. doi: 10.1186/s12903-021-01802-x (PMC8447584; doi:10.1186/s12903-021-01802-x)
Supplement: Supplementary file 4 — Additional file 4. Table S4: Forest plot for subgroup analysis. [file 12903_2021_1802_MOESM4_ESM.docx]

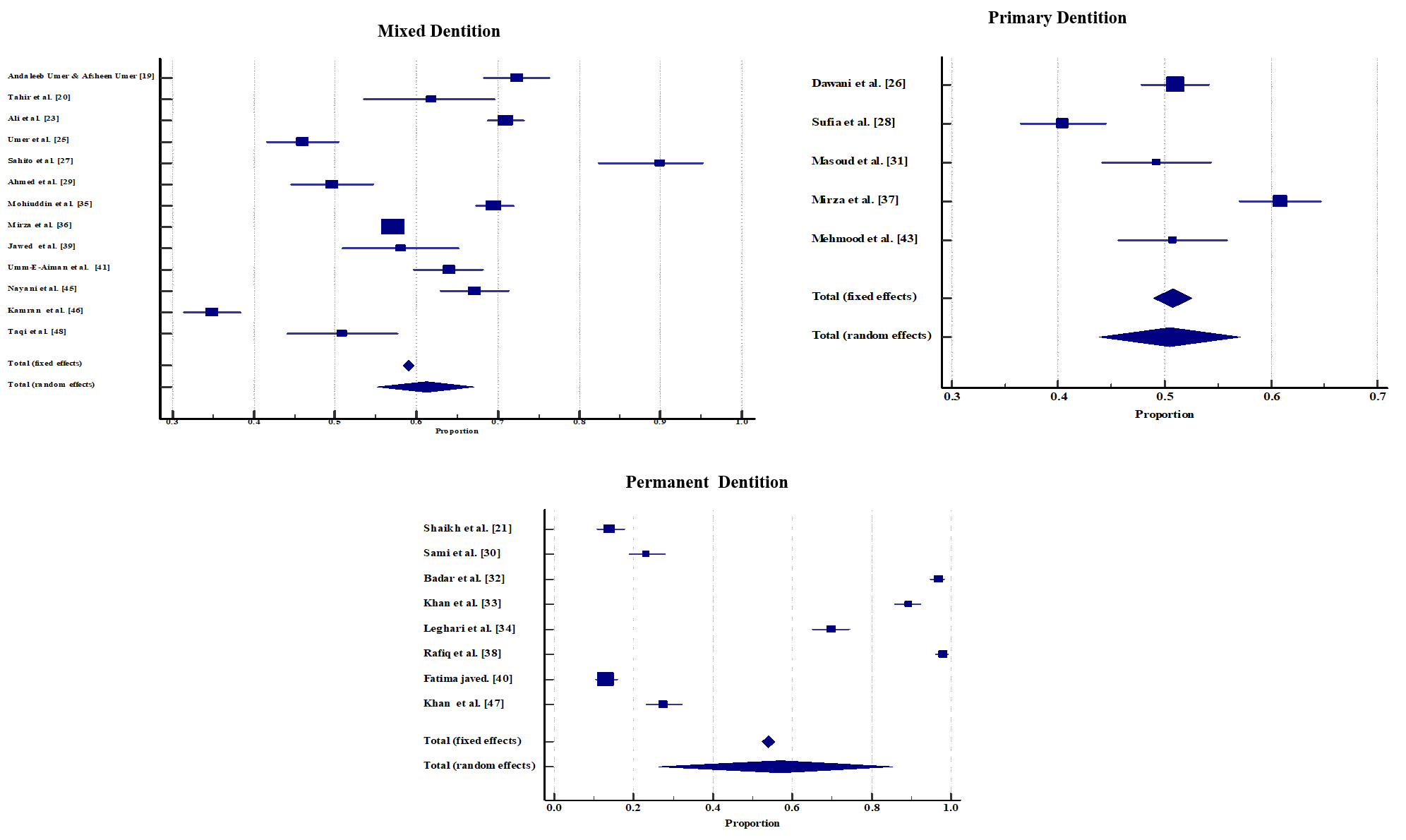


Figure S1. Forest plot showing effect of different studies and overall effect at 95 % CI regarding dental caries from (2009-2020) for primary, mixed and permeant dentitions
