# Supplementary material for: Prevalence of dental caries in Pakistan: a systematic review and meta-analysis
Source: BMC Oral Health. 2021 Sep 16;21:450. doi: 10.1186/s12903-021-01802-x (PMC8447584; doi:10.1186/s12903-021-01802-x)
Supplement: Supplementary file 5 — Additional file 5. Table S5: Funnel plot for subgroup analysis. [file 12903_2021_1802_MOESM5_ESM.docx]

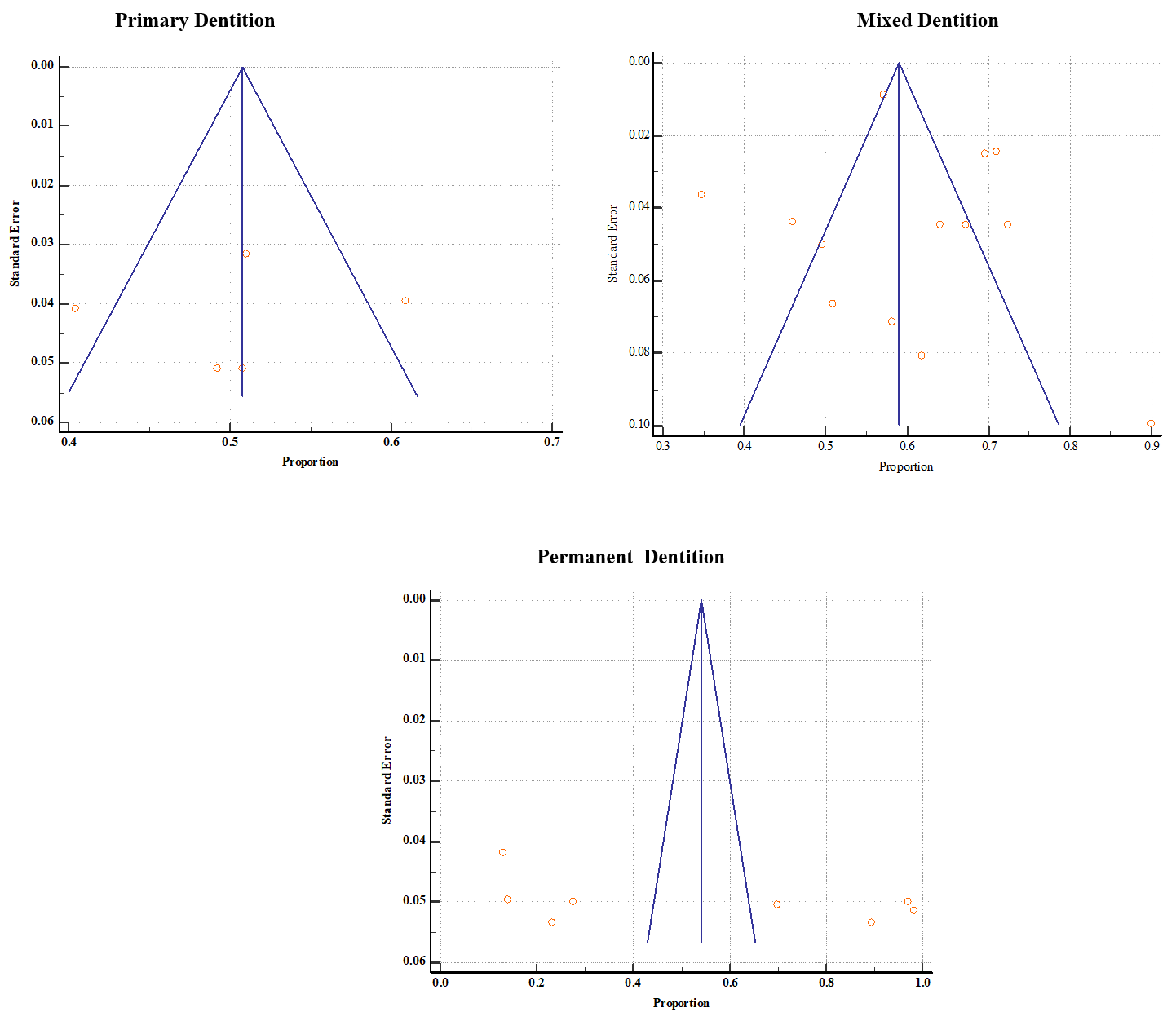


Figure S2. Funnel plot showing the prevalence of dental caries as the proportion for primary, mixed, and permeant dentitions
